# Stabilization of Influenza Vaccine Enhances Protection by Microneedle Delivery in the Mouse Skin

**DOI:** 10.1371/journal.pone.0007152

**Published:** 2009-09-25

**Authors:** Fu-Shi Quan, Yeu-Chun Kim, Dae-Goon Yoo, Richard W. Compans, Mark R. Prausnitz, Sang-Moo Kang

**Affiliations:** 1 Department of Microbiology and Immunology, and Yerkes Vaccine Center, Emory University School of Medicine, Atlanta, Georgia, United States of America; 2 School of Chemical and Biomolecular Engineering, Georgia Institute of Technology, Atlanta, Georgia, United States of America; Institut Pasteur, France

## Abstract

**Background:**

Simple and effective vaccine administration is particularly important for annually recommended influenza vaccination. We hypothesized that vaccine delivery to the skin using a patch containing vaccine-coated microneedles could be an attractive approach to improve influenza vaccination compliance and efficacy.

**Methodology/Principal Findings:**

Solid microneedle arrays coated with inactivated influenza vaccine were prepared for simple vaccine delivery to the skin. However, the stability of the influenza vaccine, as measured by hemagglutination activity, was found to be significantly damaged during microneedle coating. The addition of trehalose to the microneedle coating formulation retained hemagglutination activity, indicating stabilization of the coated influenza vaccine. For both intramuscular and microneedle skin immunization, delivery of un-stabilized vaccine yielded weaker protective immune responses including viral neutralizing antibodies, protective efficacies, and recall immune responses to influenza virus. Immunization using un-stabilized vaccine also shifted the pattern of antibody isotypes compared to the stabilized vaccine. Importantly, a single microneedle-based vaccination using stabilized influenza vaccine was found to be superior to intramuscular immunization in controlling virus replication as well as in inducing rapid recall immune responses post challenge.

**Conclusions/Significance:**

The functional integrity of hemagglutinin is associated with inducing improved protective immunity against influenza. Simple microneedle influenza vaccination in the skin produced superior protection compared to conventional intramuscular immunization. This approach is likely to be applicable to other vaccines too.

## Introduction

Influenza virus causes serious respiratory disease, affecting 5–15% of the world population annually. The dose of currently used inactivated viral or detergent split vaccines is standardized based on the content of hemagglutinin of each vaccine strain. The efficacies of the vaccines in humans are also usually evaluated by immune responses to the hemagglutinin protein [1]. The hemagglutinin content was reported to be approximately 29% of the total purified whole viral proteins [2]–[4]. The effects of hemagglutinin functional activity in the influenza vaccines on inducing protective immunity have not been well studied.

Vaccination is the most cost effective measure to prevent infectious diseases [5]. Currently licensed inactivated influenza vaccines are prepared as liquid formulations that are administered to humans intramuscularly. Vaccination exploiting the skin immune system has received great attention as an attractive immunization site [6]–[8]. Skin resident Langerhans and dermal dendritic cells are potent antigen presenting cells [9]. Some clinical studies indicated that intradermal vaccination could offer dose sparing effects, although a critical control of an equivalent low dose intramuscular immunization group was often not included [1], [10]–[14]. Belshe et al. (2007) reported a well-controlled clinical study demonstrating that intradermal immunization induced similar levels of antibody responses as intramuscular immunization [15]. Importantly, intradermal influenza vaccination was found to be effective in inducing superior immune responses in elderly adults [16], which has significant implications since 90% of the 36,000 influenza related deaths in the U.S. each year occur in seniors [17].

Previous intradermal vaccinations were performed using liquid injection devices (hypodermic needle, hollow microneedle, jet injector) [10], [16], [18]–[20]. Liquid intradermal immunizations typically require highly trained personnel and are associated with more frequent local reactions at the injection site [1], [10], [21]. Although a powder form of influenza vaccine was formulated for epidermal immunization, a special high-velocity injection device with a high antigen dose and a helium gas cylinder was required for vaccine delivery [8]. To facilitate intradermal vaccination, minimally invasive microneedle patches with a length that only penetrates across epidermis and into the superficial dermis were fabricated and used to administer small molecules and proteins into skin [22], [23].

Recently, it was demonstrated that microneedle vaccination with inactivated influenza virus in the skin could induce similar protective immunity as intramuscular vaccination [24], [25]. However, one of the challenges in microneedle vaccination is a possible loss of vaccine stability associated with the drying process during microneedle vaccine formulation. In addition, the effects of vaccine integrity as assessed *in vitro* on inducing protective immunity *in vivo* are not well investigated.

In this study, we hypothesized that vaccine integrity as represented by hemagglutination (HA) activity is a critical factor in inducing protective immune responses. We investigated the relationship between vaccine integrity and its immunogenicity, as well as the immunological differences between microneedle delivery of solid vaccine to the skin and intramuscular immunization with influenza vaccine in solution. We found that maintenance of HA activity in the vaccine was critically important in inducing isotype-switched antibodies and high levels of protective immune responses. In addition, results from this study suggest that microneedle vaccination in the skin using stabilized antigen provides insights into superior immunity against influenza infection, which might be applicable to vaccination with other antigens too.

## Results

### Microneedle-basede influenza vaccination in the skin

**Figure 1:**
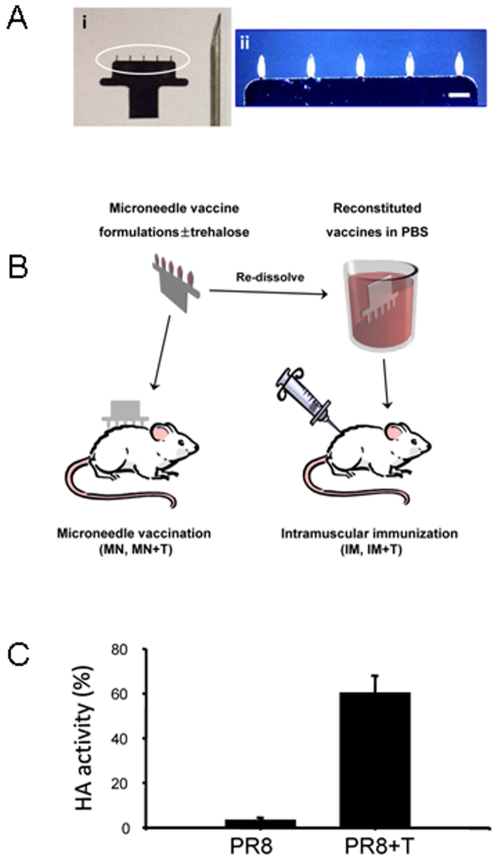
Coating of influenza vaccines on microneedles. (A) (i) Comparison of a microneedle array and hypodermic needle (23 gauge). (ii) The circled part of the microneedle array in (i) is shown as a bright-field micrograph with 5 microneedles coated with inactivated influenza virus. (B) Experimental design. Microneedles were coated with inactivated influenza A/PR8 with or without trehalose coating formulation. Some vaccine coated on microneedles with or without trehalose was dissolved in PBS for intramuscular injections. (C) HA activities (% of unprocessed control) were determined after reconstituting from microneedles coated with or without trehalose formulation.

Most vaccines including influenza are administered in liquid form using a hypodermic needle. In contrast, microneedle-based vaccination involves coating vaccine onto microneedles using a drying process, and thus represents the delivery of vaccine in a solid state (Fig. 1A). This drying process could damage antigen stability. Using formalin-inactivated whole influenza virus as a model antigen, we determined HA activity as an indicator of antigen structural integrity and vaccine stability, after coating influenza vaccines onto microneedles. As shown in Fig. 1B, vaccine coated onto microneedles was re-dissolved into PBS solution by soaking the coated needles in PBS. HA activity of the reconstituted influenza vaccine was then determined. After the drying process during coating, the influenza vaccine was found to lose most of its HA activity when reconstituted in PBS (Fig. 1B). To address this, we noted in the literature that trehalose, a disaccharide, was reported to stabilize an influenza subunit HA vaccine during freeze drying [26]. Guided by this, we found that the addition of 15% trehalose to the coating solution significantly improved the retention of HA activity of microneedle-coated vaccines up to 64% compared to an untreated vaccine (Fig. 1C). These results suggest that drying of the inactivated influenza virus has a significant damaging effect on vaccine integrity and that the addition of trehalose can largely stabilize the antigen HA activity during coating onto microneedles.

### Effects of trehalose on the immunogenicity of influenza vaccine

We next investigated the relationship between retention of HA activity of influenza vaccine and its immunogenicity after microneedle vaccination using both solid state formulations administered using MN or liquid state formulations intramuscularly. Groups of mice (n = 12) were immunized with a single dose of vaccine in the skin after coating the vaccine in the absence (MN) or presence of trehalose (MN+T) in the coating formulation (0.4 µg of viral protein). Also, to compare with the conventional intramuscular route of immunization (IM), two more groups (n = 12) were immunized with inactivated viral vaccine that was coated onto microneedles in the absence (IM) or presence of trehalose (IM+T), dissolved off the microneedles and then injected intramuscularly. A mock immunized control group with microneedle vaccination of coating buffer only in the skin was also included (n = 12).

**Figure 2:**
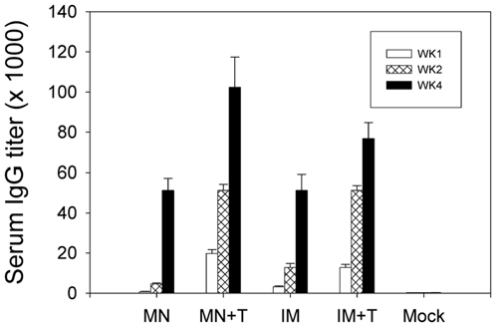
Influenza A/PR8 specific IgG responses. Mice (n = 12 per group) were immunized with microneedles coated with 0.4 µg of inactivated A/PR8 vaccine or by intramuscular injection with 0.4 µg of A/PR8 vaccine reconstituted from coated microneedles. Blood samples were collected at weeks 1, 2 and 4 after a single vaccination. Virus-specific antibody responses measured by ELISA are expressed as the endpoint titers. MN, microneedle vaccine without trehalose; MN+T, microneedle vaccine with trehalose; IM, intramuscular injection of reconstituted A/PR8 vaccine without trehalose; IM+T, intramuscular injection of reconstituted A/PR8 vaccine from trehalose-formulated microneedles; Mock, microneedles with trehalose coating buffer only (without A/PR8 vaccine). Statistical significances among groups compared are as follows: p<0.005 between MN+T and MN or IM at weeks 1, 2, and 4. p<0.05 between IM+T and IM or MN at weeks 1, 2, and 4. p<0.05 between MN+T and IM+T at week 4.

We determined vaccine (A/PR8 virus) specific total IgG and isotypes IgG1, IgG2a and IgG2b antibodies in sera at weeks 1, 2 and 4 after a single IM or microneedle vaccination in the skin. Trehalose-stabilized microneedle vaccination induced the highest levels of antibodies among the groups (Fig. 2). Trehalose stabilized vaccines (MN+T, IM+T) induced significantly higher levels of virus-specific antibodies than those in the corresponding groups without trehalose (MN, IM) at weeks 1, 2, and 4 after a single immunization (p<0.005). Among the trehalose-stabilized groups, total IgG antibody responses were higher in the trehalose microneedle group (MN+T) than those in the trehalose-stabilized IM group (IM+T) at week 4 post immunization (p<0.05). For comparison, the levels of antibody responses after intramuscular immunization with unprocessed influenza vaccine were similar regardless of the addition of trehalose to the vaccine (Table 1), which suggests that trehalose does not behave as an adjuvant, but rather acts to protect the antigen from damage during microneedle coating.

**Table 1:** Effects of trehalose on induction of immune responses to unprocessed influenza vaccine injected intramuscular^1^.

Vaccines^2^	Week 1	Week 2	Week 4
	IgG titers^3^ (×10^3^)	IgG titers (×10^3^)	IgG titers (×10^3^)
PR8i	15.2±5	51.2±20	102.4±28
PR8i + Trehalose	12.8±3	60.8±25	90.4±20
Mock	0.1±0.05	0.1±0.05	0.1±0.05

1 Virus specific total IgG antibody responses were determined at weeks 1, 2, and 4 after a single immunization of mice (6 BALB/c mice for each group) with inactivated influenza vaccine with or without trehalose.

2 Vaccines are unprocessed inactivated whole virus (A/PR8i, 0.4 µg), unprocessed inactivated virus plus trehalose (A/PR8i 0.4 µg + 15% trehalose), or mock (15% trehalose).

3 Virus specific total IgG antibody titers were expressed as the highest dilution having a mean optical density at 450 nm greater than the mean value plus 3 standard deviations of naïve serum samples.

**Figure 3:**
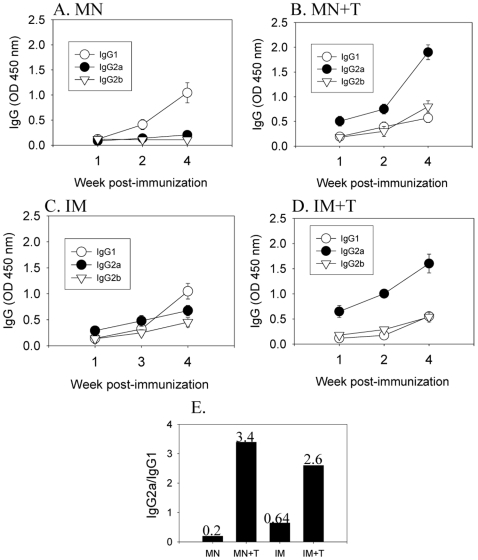
Effects of trehalose stabilized vaccine on antibody isotype responses. (A) MN group, (B) MN+T group, (C) IM group, and (D) IM+T group. Kinetics of A/PR8 virus specific isotype antibody responses (IgG1, IgG2a, IgG2b) were determined at weeks 1, 2 and 4 after a single vaccination. Results are expressed as averages of optical density readings at 450 nm (OD_450_) with 100-fold diluted serum samples in each group of mice (n = 12). (E) Ratios of IgG2a/IgG1 based on optical density readings with week-4 samples. Groups are described in the legend of Fig. 2.

The pattern of antibody isotypes indicates either T helper type 1 (Th1) or type 2 (Th2) like immune response corresponding to dominance of the IgG2a or IgG1 isotype antibody, respectively [27]. When isotype-switched antibodies were examined, we found a very striking pattern among the groups studied (Fig. 3 and Table 2). The ratios of IgG2a to IgG1 (IgG2a/IgG1) were analyzed based on isotype antibody levels determined by optical densities (Fig. 3E) and end-point dilution titers (Table 2). Both analyses showed similar results. The trehalose-stabilized vaccines (MN+T, IM+T) induced IgG2a as the dominant isotype (Fig. 3B, 3D, 3E, and Table 2). A similar pattern of IgG2a dominant immune responses was previously observed in mice intramuscularly immunized with intact influenza vaccine [28], [29]. In contrast, trehalose-negative vaccine groups (MN, IM) showed IgG1 as the dominant isotype antibody (Figs. 3A, 3C), which resulted in a switch in IgG1/IgG2a ratio of >1 for trehalose-containing groups (IM+T, MN+T) to <1 for groups without trehalose (IM, MN). Therefore, these results suggest that the retention of HA activity by trehalose in the influenza vaccine significantly affected the magnitude of humoral immune responses as well as the pattern of isotype switching.

**Table 2:** IgG1 and IgG2a titers (×10^3^) in serum before and after challenge^1^.

Group^2^	IgG1		IgG2a	Ratio^3^ (IgG2a/IgG1)	
	Before	After	Before	After	Before	After
MN	51.2±6	102.4±15.5	6.4±1	12.8±2	0.12	0.12
MN+T	25.6±5	76.8±8.1	102.4±20	204.8±30	4.0	2.7
IM	12.8±2	19.2±4	9.6±2	12.8±5	0.75	0.67
IM+T	9.6±1	6.4±1	25.6±3	25.6±5	2.6	3

1 Virus specific IgG1 and IgG2a antibody responses were determined and compared among groups before challenge (at week 4 post immunization) and after challenge (at day 4 post challenge). Antibody titers were expressed as the highest dilution having a mean optical density at 450 nm greater than the mean value plus 3 standard deviations of naïve serum samples.

2 MN, microneedle vaccine without trehalose; MN+T, microneedle vaccine with trehalose; IM, reconstituted A/PR8 vaccine without trehalose; IM+T, reconstituted A/PR8 vaccine from trehalose-formulated microneedles; Mock, microneedle with trehalose coating buffer only (without A/PR8 vaccine).

3 Isotype antibody ratios (IgG2a/IgG1) based on antibody titers before challenge (Week 4) and after challenge (Day4). Before, serum samples (n = 12) collected at week 4 after a single immunization; After, serum samples (n = 6) collected at day 4 after challenge.

### Microneedle vaccine with higher HA activity enhances functional antibodies

**Figure 4:**
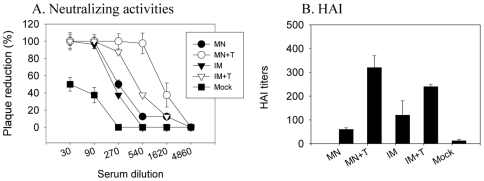
Trehalose stabilized vaccine enhances functional antibody responses. Serum neutralizing titers (A) and hemagglutination inhibition (HAI) titers (B) were determined at week 4 after a single vaccination (n = 12). Neutralizing activities were expressed as the percentage of plaque reduction compared to a naïve serum control. Significant differences were found among groups of mice: For neutralizing titers at the 540 dilution for plaque reduction, p<0.005 between MN+T and MN or IM. At the serum dilution 270, p<0.05 between IM+T and IM or MN. For HAI titers, p<0.001 between MN+T and MN or IM, and p<0.05 between IM+T and IM or MN.

Levels of functional antibodies measured as hemagglutination inhibition (HAI) and/or virus neutralizing activities in immune sera are known to be better immune correlates for protection [1], [30]. As shown in the Fig. 4, highest titers of HAI and neutralization activity were induced by microneedle vaccination in the skin using stabilized vaccine (MN+T). As shown in Fig. 4A, mice immunized with the trehalose-stabilized vaccines showed considerably higher neutralizing activities, showing a 6-fold comparing MN and MN+T groups (270 vs 1620 titers of 50% plaque reduction, p<0.005) and 2-fold between IM and IM+T groups (270 vs 540 titers of 50% plaque reduction, p<0.05). Similarly higher HAI titers were observed in the trehalose-stabilized vaccine compared to corresponding groups without trehalose in the formulation (Fig. 4B, p<0.001 between MN and MN+T; p<0.05 between IM and IM+T). These results indicate that microneedle delivery of stabilized vaccines can be superior to IM immunization in inducing neutralizing and hemagglutination inhibiting antibodies, and that maintaining HA functional activity in the influenza vaccine is critical to inducing high levels of functional antibodies.

### Maintaining HA activity of influenza vaccines improves the efficacy of protection

**Figure 5:**
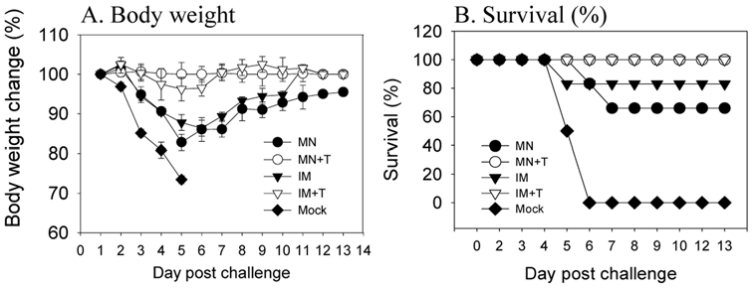
Trehalose stabilization of vaccine improves protective efficacy. At week 5 after a single vaccination, mice (n = 12) were challenged with a lethal dose (A/PR8 virus, 20× LD50) and were monitored daily to record body weight changes (A) and survival rates (B). Groups of mice are described as in the legend of Fig. 2.

To determine whether vaccine stability measured by HA activity or the routes of immunization (i.e., microneedle versus intramuscular) affects protective immunity, vaccinated mice including a mock control were challenged with a lethal dose of A/PR8 virus (20× LD_50_) at 5 weeks after a single microneedle vaccination in the skin or intramuscular immunization (Fig. 5). All mock-immunized control mice rapidly lost body weight and died by day 6 post lethal-challenge. Groups of mice immunized with either inramuscular or microneedle vaccination without trehalose formulation (MN and IM) showed significant illness as determined by loss in body weight up to 15 to 17% whereas the intramuscular group with trehalose (IM+T) showed a only transient body weight loss of 5%. Importantly, the trehalose-microneedle group (MN+T) did not show any loss in body weight. As expected from body weight changes, survival rates of groups without trehalose were lower (70% for MN and 80% for IM) than those groups with trehalose, which showed 100% survival (MN+T, IM+T). Therefore, these results support the conclusion that stabilization of influenza vaccine by trehalose during microneedle coating is critically important for inducing protective immunity.

### Microneedle delivery of stabilized vaccine provides effective viral control

**Figure 6:**
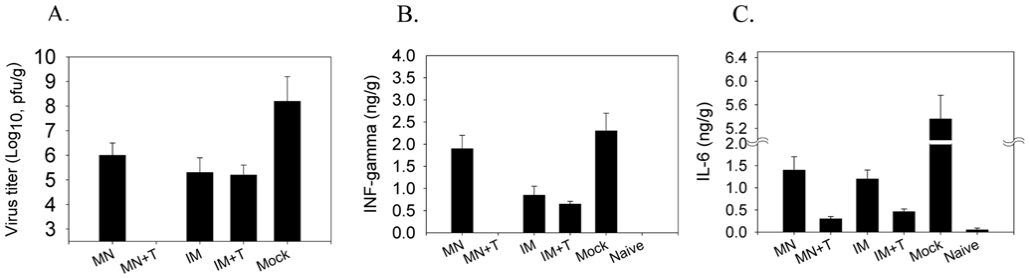
Trehalose-stabilized microneedle vaccine improves control of lung viral replication. Lungs from individual mice in each group (n = 6 out of 12 mice per group) were collected on day 4 post-challenge, weighed, and extracted in media. Slight variations in lung tissue weight recovered from individual mice were adjusted (0.25 g lung tissue/ml). Virus titers (plaque forming units, pfu) in log10 (A), or IFN- γ (B) and IL-6 (C) cytokines in nano-grams (ng) per gram (g) lung tissue are expressed as geometric mean values. IFN-γ and IL-6 in lung extracts were determined by ELISA. Groups of mice are described in the legend of Fig. 2. Naïve is an uninfected mouse control.

To better appreciate the efficacy of protection, we determined the viral titers and inflammatory cytokine levels in lungs at day 4 post challenge infection. The groups of mice with microneedle or intramuscular vaccination (MN, IM) had lower lung viral titers by 20- and 75-fold compared to those in the mock-immunized control (p<0.01), but still showed lung viral titers in a range of from 2.9×10^5^ to 1×10^6^ pfu/g lung tissue (Fig. 6A). The IM immunized group with trehalose (IM+T) showed lung viral titers of 2.4×10^5^ pfu/g lung tissue, 100-fold lower compared to the mock control (Fig. 6A, p<0.005). Levels of lung viral titers in the group of microneedle delivery with trehalose (MN+T) were lowest and were under the detection limit (<270 pfu/g lung tissue).

Acute infection by pathogenic influenza virus causes severe lung inflammation [31], [32]. Thus, levels of inflammatory cytokines can be used as an additional parameter in evaluating protective efficacy. The control mice showed the highest levels of interferon-γ (IFN-γ) and interleukin-6 (IL-6) pro-inflammatory cytokine levels in lungs at day 4 post challenge. Microneedle delivery of trehalose stabilized vaccine (MN+T) was found to be most effective in lowering inflammatory cytokines whereas high levels of inflammatory cytokines were detected in the trehalose-negative microneedle group (MN), showing a correlation between lung viral titers and inflammatory cytokines (Fig. 6B and 6C). Also, groups of mice immunized intramuscularly with trehalose-stabilized vaccine (IM+T) showed moderate or low levels of cytokines compared to the trehalose-negative groups (p<0.05) (Fig. 6B and 6C). In summary, these results show that microneedle delivery of stabilized influenza vaccine induced superior protective immunity in controlling lung viral replication of challenge virus compared to microneedle delivery of unstabilized vaccine and to intramuscular immunization using stabilized or unstabilized vaccine.

### Stabilized microneedle vaccine provides rapid recall immune responses

**Figure 7:**
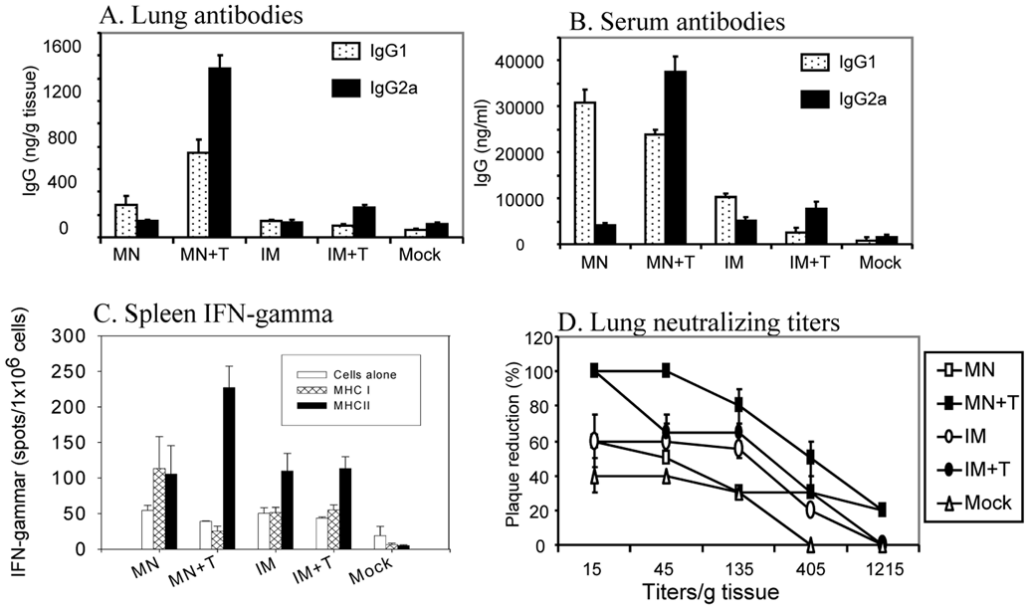
Higher recall immune responses induced by trehalose stabilized microneedle vaccine. Lung, serum, and spleen samples were collected from individual mice in each group (n = 6) at day 4 post challenge. (A) Virus specific IgG1 and IgG2a antibodies in lungs (ng/g tissue: ng antibodies per g lung tissue). (B) Virus specific IgG1 and IgG2a antibodies in sera (ng/ml: ng antibodies per ml sera). (C) IFN- γ secreting splenocytes (spots/1×10^6^ spleen cells) after stimulation with hemagglutinin-specific MHC I and II peptides. (D) Neutralizing activities in lung. Serial dilutions of lung samples were incubated with infectious influenza viruses and percentiles of plaque forming units were determined. Titers/g tissue: dilution factors per g lung tissue from the lung extracts (0.25 g/ml). Groups of mice are the same as in Fig. 5. Significant differences were found among the groups: For lung IgG2a antibody (A), p<0.001 between MN+T and other groups; for lung IgG1 (A), p<0.001 between MN+T and IM or IM+T. For serum IgG2a antibody (B), p<0.001 between MN+T and other groups; for IgG1 (B), p<0.005 between MN and IM or IM+T, p<0.05 between MN+T and IM or IM+T. For spleen IFN-γ secreting cells (C), p<0.05 between MN+T and other groups. For lung neutralizing titers, p<0.05 between MN+T and other groups at 15× dilution.

To better understand the significantly improved viral clearance in the hemagglutinin-stabilized microneedle group (MN+T), we determined virus-specific antibody levels in lungs and sera at day 4 post challenge infection (Fig. 7). Interestingly, the group of mice that received microneedle vaccine formulated with trehalose (MN+T) showed highest levels of both IgG1 and IgG2a antibodies specific to virus in lungs at day 4 post challenge infection (p<0.001) (Fig. 7A). Microneedle vaccination without trehalose (MN) induced moderately high levels of IgG1 antibody as a dominant isotype in this group. In contrast, inramuscular immunized groups (IM, IM+T) did not induce significant levels of recall antibody responses specific to virus in lungs.

Higher levels of both recall serum IgG1 and IgG2a antibodies were also observed in the group immunized by microneedle delivery with trehalose-formulated vaccine (MN+T) than those in the corresponding inramuscular immunization groups (p<0.05 between MN+T and IM+T for both IgG1 and IgG2a) (Fig. 7B and Table 2). Increases in serum IgG1 antibodies were also prominent in the group immunized by microneedle delivery after challenge infection (p<0.005 between MN and IM or IM+T for IgG1, Fig. 7B).

To determine T cell responses, spleen cells were harvested from immunized mice at day 4 post challenge infection. After *in vitro* stimulation of splenocytes with A/PR/8/34 hemagglutinin-specific MHC I or MHC II peptides, cytokine secreting cells were quantified (Fig. 7C). The microneedle vaccine formulated with trehalose (MN+T) showed the highest levels of MHC II peptide-specific IFN-γ secreting splenocytes (p<0.05 among the immunized groups). In the inramuscular groups (IM, IM+T), there were no significant differences in levels of IFN-γ secreting splenocytes with or without trehalose formulation.

The lung is the primary site for replication of influenza virus, and neutralizing antibodies in lungs are expected to play a significant role in clearing the viruses. Thus, neutralizing titers were determined in lung samples collected at day 4 post challenge (Fig. 7D). The highest neutralizing titers of 45 with 80% plaque reduction were detected in lung samples after microneedle vaccination with trehalose formulation (MN+T), which was more than 3-fold higher compared to those in the corresponding unstabilized or other groups. Without trehalose stabilization, both inramuscular and microneedle groups (IM, MN) showed lower levels of neutralizing titers in lungs. These lung neutralizing titers showed correlations with lung viral titers post challenge as shown in Fig. 6A. These results suggest that microneedle delivery of vaccine stabilized by trehalose is superior in inducing rapid recall mucosal and systemic immune responses upon challenge infection compared to microneedle delivery of unstabilized vaccine and to intramuscular immunization using stabilized or unstabilized vaccine.

## Discussion

In the present study, we investigated microneedle vaccination via the skin and the effects of loss of HA activity of the influenza vaccine on its immunogenicity. Coating microneedles with inactivated influenza virus caused loss of HA activity of the vaccine whereas addition of trehalose to the coating buffer significantly improved the maintenance of HA activity. Immunization of mice with unstabilized influenza vaccine via microneedle or intramuscularly showed significantly lower levels of IgG2a antibody, and decreased levels of functional antibodies and protective efficacies compared to those induced by trehalose-stabilized influenza vaccines. These results indicate the importance of maintaining influenza vaccine HA activity in inducing protective immunity. Also, immune responses were different in quantities as well as qualities depending on the HA activities of influenza vaccine and the route of vaccine delivery. Particularly, microneedle vaccination in the skin was significantly more effective than intramuscular immunization in controlling challenge virus replication and in inducing recall immune responses.

Recent studies reported that microneedle immunization with 10-µg inactivated influenza virus provided comparable immune responses and protection as the conventional intramuscular immunization, although stabilities of the microneedle vaccines were not investigated [24], [25]. We found that the stability of influenza vaccines significantly influenced the pattern of antibody isotypes, indicating the types of immune responses: IgG1 and IgG2a isotypes are indicative of T helper type 2 (Th2) and type 1 (Th1), respectively. The quality of the immune response induced by vaccination also seems to be important for inducing protective immunity. Natural virus infection is known to induce IgG2a isotype as the dominant antibody [33]–[35]. The Fc domain of IgG2a antibody interacts more efficiently with complements in serum and Fc receptors on immune cells resulting in activation of the complement system, antibody-mediated cellular cytoxicity, and clearance of opsonized virus by macrophages [36], [37], [38]–[41]. Consistent with previous studies, this study suggests that the induction of high levels of IgG2a antibodies together with IgG1 antibodies by stabilized microneedle vaccination contributed to effective control of challenge virus replication.

One of the interesting observations from the present study is the fact that the functional integrity of hemagglutinin is a critical determining factor for inducing IgG2a isotype antibody after microneedle or intramuscular delivery of vaccine as well as for inducing antibody responses to influenza. Also, trehalose itself was found not to significantly affect host immune responses to influenza, and therefore does not appear to play a significant role as an adjuvant. On this basis, it is likely that the major role of trehalose formulation is to stabilize HA activity and thereby maintain influenza vaccine integrity.

The functional integrity of hemagglutinin in the influenza vaccine might be important for effective interactions with receptors of target cells such as Langerhans cells, dermal dendritic cells, keratinocytes, and/or other immune cells. Receptor-mediated entry of whole inactivated influenza vaccines into the target cells can deliver their single-stranded RNA molecules, an activator for toll-like receptor-7 (TLR7), resulting in the induction of Th1 type immune responses [9], [42]. However, the expression of TLR7 was observed in keratinocytes and in certain lineages of dendritic cells but not in the Langerhans cells [42], [43], indicating that the TLR7 signaling pathway is not the main contributor for activating skin resident Langerhans cells. An alternative possibility is that immune cells were activated by interactions between intact hemagglutinin of vaccines with sialic acid or other receptors on immune cells via an activation pathway independent of TLR7. A recent study demonstrated that fusion activity of inactivated influenza vaccine was required for its superior immunogenicity to split and subunit vaccines and that the immune responses could be mediated by either TLR signaling dependent or independent activation of the innate immune system [42].

Our data suggest that the functional integrity of hemagglutinin in the influenza vaccine may influence the types of antigen presenting cells. Th1 type immune responses are known to be induced most likely by pathogen interactions with receptors on host innate immune cells [42]–[45]. Some dendritic cells are more likely to induce Th1 type immune responses whereas macrophage cells induce Th2 type responses affecting the pattern of antibody isotypes [46]. In comparing groups with and without trehalose-mediated HA stabilization of influenza vaccine, microneedle groups showed 6- to 9-fold differences in HAI and neutralizing titers (MN versus MN+T) whereas intramuscular groups showed approximately 2-fold differences in functional antibody induction (IM versus IM+T). Thus, maintenance of influenza vaccine HA activity had a greater impact on microneedle vaccination in the skin. The underlying mechanisms how the immune system differentially induces immune responses to influenza vaccines depending on the vaccine HA activities remain to be determined.

Intradermal influenza vaccination has attracted widespread interest. However, detailed immunologic studies have not been carried out. Vaccines delivered intradermally were effective against rabies, BCG, hepatitis B, and influenza antigens [15], [47]. These vaccines were delivered in liquid formulations using hypodermic needles, hollow microneedle, or jet injector devices. In contrast, our study used microneedles coated with inactivated virus in a dry state for vaccination to the skin. Use of solid state vaccine may affect antigen uptake and presentation, in addition to providing a stable formulation that does not require reconstitution before administration.

Microneedle-based delivery differs from intramuscular injection not only in the route of administration, but also in the preparation of the vaccine. To isolate out the effects of potential antigenic changes during microneedle vaccine preparation independent of the route of administration, we reconstituted vaccines from coated microneedles and injected them intramuscularly for comparison of immunogenicity and protective efficacies with microneedle vaccination to the skin. Using a trehalose formulation, microneedle vaccination in the skin was significantly more immunogenic than intramuscular immunization as evidenced by a range of different immunologic assays: binding and functional antibodies, post-challenge lung viral titers and inflammatory cytokine levels, and recall mucosal and systemic responses to influenza. Among the range of immunologic data, the recall immune responses to influenza in the microneedle group (MN+T) with a single vaccination to the skin were significantly stronger than intramuscular immunization (IM+T). After taking up intradermally delivered antigens, skin-derived dendritic cells are known to migrate to the systemic and mucosal compartments [48]–[50], which might be involved in rapid recall immune responses after microneedle vaccination in the skin as demonstrated in this study. Therefore, our detailed immunologic study provides deeper explanations for potential improved protective efficacies by vaccine delivery to the skin.

In summary, this study demonstrates that the integrity of influenza vaccine as represented by HA activities is a critically important factor for determining antibody isotypes, inducing functional antibodies, and providing effective protective immunity as well as recall immune responses to influenza. Also, our study provides insight into formulating a vaccine maintaining structural and conformational integrity through the use of trehalose as a stabilizer. Immunologic data from this study offers a partial explanation for improved protection by delivering vaccines to the skin. Finally, the results show that a solid formulation of an enveloped virus antigen with biologically active glycoproteins can be at least as effective as a liquid form of vaccine if an optimized stabilizer is used. In addition to possible immunologic advantages, solid microneedle vaccination may offer logistic advantages such as possible self-administration, less dependence on a cold-chain and highly trained medical personnel, and less pain compared to the conventional intradermal or intramuscular delivery of liquid form vaccines.

## Materials and Methods

### Preparation of influenza virus

Influenza virus, A/PR/8/1934 (H1N1, abbreviated as A/PR8), was grown in 10-day old embryonated hen's eggs and purified from allantoic fluid by using a discontinuous sucrose gradient (15%, 30% and 60%) layers. The purified virus was inactivated by mixing the virus with formalin at a final concentration of 1∶4000 (v/v) as previously described [51], [52]. Inactivation of the virus was confirmed by plaque assay on confluent monolayer Madin-Darby canine kidney (MDCK) cells and inoculation of the virus into 10-day old embryonated hen's eggs. Inactivated whole virus vaccine (A/PR8) was used to coat solid metal microneedles for vaccination in the skin. For use in challenge experiments, mouse adapted A/PR8 virus was prepared as lung homogenates from mice infected 4 days earlier [53], [54].

### Microneedle vaccine preparation

Stainless steel microneedles of approximately 700 µm in length (Fig. 1A) were fabricated by laser cutting and electro-polishing technology as described previously [23]. A manual dip-coating device was used to coat a vaccine onto microneedles by dipping and air-drying six times at 25°C [23], [55]. The vaccine coating solution was composed of 1% (w/v) carboxymethylcellulose sodium salt (Carbo-Mer, San Diego, CA), 0.5% (w/v), Lutrol F-68 NF (BASF, Mt. Olive, NJ), and inactivated virus (A/PR8, 1 mg/ml based on total protein contents) in phosphate-buffered saline (PBS) with or without 15% (w/v) trehalose (Sigma Aldrich, St. Louis, MO). Half of the microneedles coated with inactivated virus vaccine were de-coated to reconstitute vaccines in PBS for intramuscular immunization controls and the other half were used for microneedle immunization (Fig. 1B).

To determine the amount of inactivated virus vaccine coated on microneedles, vaccine-coated microneedles were incubated in PBS for 12 h at 4°C and the total protein content of the reconstituted vaccine from an array of five microneedles was measured by a DC protein assay kit (Bio-Rad, Irvine, CA). The retained HA activities were also determined in the reconstituted vaccines. Dissolved vaccines in PBS (50 µl) was serially diluted in 50 µl of PBS mixed with an equal volume of a fresh 0.5% suspension of chicken red blood cells (Lampire Biological Laboratories, Pipersville, PA) and incubated for 1 h at 25°C. The titers were determined as the endpoint dilutions inhibiting the precipitation of red blood cells.

### Immunization and challenge infection

Female inbred BALB/c mice (Charles River, Wilmington, MA) aged 6 to 8 weeks were used. Groups of mice (12 mice per group) were immunized with coated microneedle vaccine (0.4 µg total protein) for delivery to the skin or reconstituted vaccines (0.4 µg protein/100 µl) for intramuscular immunization in the upper quadriceps muscles of mice (both legs each with 50 µl). The four groups of immunized mice were designated as trehalose-negative formulated microneedle vaccine (MN), trehalose-positive formulated microneedle vaccine (MN+T), trehalose-negative reconstituted vaccine (IM) and trehalose-positive reconstituted vaccine (IM+T) for intramuscular injection. For microneedle delivery, mice were anesthetized with ketamine (110 mg/kg, Abbott Laboratories, Chicago, IL) mixed with xylaxine (11 mg/kg, Phoenix Scientific, St. Joseph, MO). Hair on the dorsal surface of mice was removed by a hair-removing cream (Nair, Church and Dwight Company, Princeton, NJ) with a moisturized cotton stick. After cleaning with a soaked cotton ball (70% ethanol) and drying with a hair dryer, an array of vaccine-coated microneedles was inserted into the skin and left in place for 10 min for release of the vaccine antigen from the microneedle.

For challenge infections, mice lightly anesthetized with isoflurane were intranasally infected with a lethal dose of A/PR8 virus (20× LD50) in 50 µl of PBS at 5 weeks after a single dose immunization. Mice were observed daily to monitor changes in body weight and to record mortality. Mice with >25% loss of body weight were sacrificed to prevent undue suffering. These studies were approved by Emory University IACUC.

### Antibody responses and hemagglutination inhibition (HAI) titer

Influenza virus-specific antibodies of different isotypes (IgG, IgG1, IgG2a and IgG2b) were determined by enzyme-linked immunosorbent assay (ELISA) plates coated with A/PR8 viral antigen and by using anti-mouse IgG isotype specific secondary antibodies as described previously [30], [54]. Antibody concentrations (ng per g lung tissues or ng per ml sera) were determined using standard curves for mouse IgG1 and IgG2a antibodies. For determination of hemagglutination-inhibition (HAI) titers, serum samples were first treated with receptor destroying enzyme (Denka Seiken, Kayabacho,Chuo-ku,Tokyo) by incubation overnight at 37°C, and then incubated 30 min at 56°C. Sera were serially diluted, mixed with 4 HA units (HAU) of influenza A/PR8 virus, and incubated for 30 min at room temperature prior to adding 0.5% chicken red blood cells. The highest serum dilution preventing hemagglutination was scored as the HAI titer as described [30].

### Neutralization, lung viral titer and lung inflammatory cytokine assays

Virus neutralization assay was performed using MDCK cells (American Type Culture Collection, VA, USA) following a previously described procedure [30], [54]. The neutralization activity was expressed as the percentage of plaque reduction. Lung viral titers at day 4 post challenge were determined by counting plaques formed on the MDCK cells as previously described [30], [54]. Inflammatory cytokines (IL-6, IFN-γ) in lungs collected at day 4 post challenge were analyzed by Ready-Set-Go cytokine kits (eBioscience, San Diego, CA) following the manufacturer's procedure as previously described [54].

### Virus-specific recall immune responses upon virus challenge

Recall immune responses were determined from serum, lung, and spleen at day 4 post-challenge. To determine T cell responses, spleen cells were harvested from immunized and mock control mice at day 4 post-challenge and were used to determine cytokine producing T cell responses by ELISPOT as previously described [54]. Briefly, spleen cells (10^6^ cells per well of 96 well plates) were stimulated *in vitro* with A/PR8 hemagglutinin specific peptides [56], [57]; a mixture of two major histocompatibility complex class I (MHC-I) peptides (IYSTVASSL and LYEKVKSQL) or a pool of five MHC-II peptides (SFERFEIFPKE, HNTNGVTAACSH, CPKYVRSAKLRM, KLKNSYVNKKGK, and NAYVSVVTSNYNRRF). Regents for cytokine ELISPOT were purchased from BD/PharMingen (San Diego, CA).

### Statistics

All parameters were recorded for individuals within all groups. A two-tailed Student's t-test was performed when comparing two different conditions. Statistical comparisons of three or more conditions were carried out using the correlation and regression test of the PC-SAS system (SAS Institute, Cary NC). A *P* value less than 0.05 was considered to be significant.
